# Telemedicine for Musculoskeletal Care During the COVID-19 Pandemic: Evaluating Readiness of Saudi Citizens

**DOI:** 10.7759/cureus.13380

**Published:** 2021-02-16

**Authors:** Naif M Alhamam, Rayan A Buhalim, Ibrahim H Almakhayitah, Abdulelah W AlBahr, Ibrahim A AlYaeesh

**Affiliations:** 1 Orthopaedics, King Faisal University, Al Ahsa, SAU; 2 Medicine, King Faisal University, Al Ahsa, SAU

**Keywords:** telemedicine, orthopedic, saudi arabia, musculoskeletal, knowledge, corona virus, e-health, covid-19

## Abstract

Introduction: Since coronavirus disease 2019 (COVID-19) was announced as a global pandemic, it has become important to control the pandemic with several approaches, including limiting hospital visits. Telemedicine is a good option to help reduce in-person visits during the pandemic. Saudi Arabia has prepared for this pandemic by implementing applications, such as Tetamman and Seha. In this study, we aimed to determine the readiness of the Saudi population to use telemedicine for musculoskeletal care during the COVID-19 pandemic.

Methods: A cross-sectional study was conducted from July 2020 to October 2020. The study used a predesigned, self-administered questionnaire with acceptable internal consistency (Cronbach’s α=0.79). A questionnaire with 30 questions was distributed electronically and randomly to the Saudi population. The included participants were Arabic speakers, Saudis, and men or women age 18 years or older.

Results: A total of 635 respondents, of which 250 were men (39.4%) and 385 were women (60.6%), completed the questionnaire. The most common region of residence was the Central region (41.6%), followed by the Eastern region (28%). The proportions of patients who had knowledge about virtual clinics and who used a virtual clinic during the COVID-19 pandemic were 47.6% and 30.4%, respectively. The mean overall attitude score was 24.4 (standard deviation, 9.9) of 35 points; negative, neutral, and positive attitudes were reported among 9.9%, 54.3%, and 35.7% of respondents, respectively. Compared with the older population, younger-aged participants (≤25 years) had significantly more positive attitudes about virtual clinics (χ^2^=6.068; p=0.048). Those respondents who had never been married showed significantly more positive attitudes about virtual clinics compared with those who had been married (χ^2^=6.695; p=0.035).

Conclusions: The studied Saudi population shows a moderate level of acceptance of the concept of using telemedicine in musculoskeletal conditions, but some issues about patient access and understanding of the technology remain unaddressed.

## Introduction

On March 11, 2020, the World Health Organization announced that coronavirus disease 2019 (COVID-19) had become a global pandemic [1]. To prevent and control the spread of the rapidly evolving virus, global health agencies considered several precautions, such as suspension of all nonurgent elective surgeries, limitation of clinical visits in hospitals, and other measures to decrease the risk of cross-infection [2,3]. Without clinical visits in the health facilities, another system to treat patients during the COVID-19 pandemic is necessary. One of the most effective ways to continue healthcare delivery in pandemic circumstances is telemedicine [4]. Telemedicine is defined as the delivery of healthcare to patients at a distance without the need to come to the healthcare facility [5]. This technology offers many benefits; it can provide care that may not be an option otherwise, improve the quality of screening programs, reduce healthcare costs, and more. Conversely, some challenges associated with telemedicine include a breakdown in the doctor-patient relationship and patient doubts about health information received virtually [6]. Telemedicine has been used in the surgical field to improve the practice of surgeons in treating their patients [6]. During the COVID-19 pandemic, orthopedic surgeons should reach patients without face-to-face consultations [7]. In Saudi Arabia, the Ministry of Health provided some applications to implement telemedicine in the country. Patients affected by COVID-19 can receive medical care through these applications. One application, Rest Assured or Tetamman, provides healthcare support mainly to citizens or residents in domestic isolation or quarantine [8]. Another application is Seha which offers consultations and diagnostic services to patients [9]. Telemedicine users have reported general satisfaction with this modality in terms of effectiveness and efficiency [10]. The COVID-19 pandemic has driven providers to find alternative methods to deliver safe orthopedic healthcare to patients. Telemedicine can provide constant continuous care for orthopedic practice [11,12]. To the best of our knowledge, no study has been conducted to determine the perception about telemedicine and readiness of patients to use telemedicine for musculoskeletal conditions during the COVID-19 pandemic in Saudi Arabia. The aim of our study is to determine the readiness of the Saudi population to use telemedicine for musculoskeletal care during the COVID-19 pandemic.

## Materials and methods

Study design and participants

A cross-sectional study was conducted from July 2020 to October 2020 in Saudi Arabia. A minimum of 385 participants needed to be recruited from the general population of Saudi Arabia to achieve a 95% confidence interval and a 5% margin of error [13,14]. A self-administrated online questionnaire was distributed randomly irrespective of their musculoskeletal condition. The included participants were Arabic speakers, Saudis, and men or women who are aged 18 years or older. Ethical approval was obtained from the ethical committee of the College of Medicine at King Faisal University (2020-10-24). Informed consent was taken from all participants before their commencement of the questionnaire, and their personal information was kept confidential.

Data collection

The sampling technique of this study was the convenience random sampling method where an Arabic online questionnaire was distributed to the community using social media platforms such as WhatsApp and Twitter. The questionnaire was taken from a previous study. The research idea and participant roles were explained and the participants' consent was obtained. Before distributing the survey, we tested the validity of the questions in a pilot study of 20 participants. Internal consistency was acceptable (Cronbach’s α=0.79). The questionnaire was divided into four parts: (1) sociodemographic data (11 questions), (2) questions about musculoskeletal care (4 questions), (3) questions about telemedicine readiness (7 questions), and (4) knowledge and acceptance of telemedicine during the COVID-19 pandemic (8 questions) [15].

Calculation of scores

The attitude toward the use of a virtual clinic or telemedicine was drawn from seven statements with a 5-point Likert scale; categories ranged from “strongly disagree,” coded as 1, to “strongly agree,” coded as 5 [15]. The total score was calculated by adding points from the seven questions. The scores generated a minimum of 7 points and a maximum of 35 points; higher scores reflected more positive attitudes toward virtual clinic use. To determine the attitudes of respondents toward the use of virtual clinics, total scores were classified as follows: attitude scores of 7-17 points reflected negative attitudes; 18-26 points, neutral; 27-35 points, positive.

Data analysis

Data were reported as the frequency, percentage, or mean and standard deviation, as appropriate. Chi-square tests were applied for between-group comparisons. All statistical analyses were performed using the Statistical Package for the Social Sciences (SPSS) software for Windows (version 21; IBM, Armonk, NY). P-values of <0.05 were considered statistically significant.

## Results

We recruited 635 patients across Saudi Arabia to evaluate readiness for a virtual clinic. As seen in Table 1, the most common age group was 18-25 years (55.7%); more than 60% were women, and more than two-thirds (68.7%) had a bachelor’s degree or higher. The majority of participants were never married (69.3%). The most common region of residence was the Central region (41.6%), followed by the Eastern region (28%). Nearly, all participants resided in the city (88.3%), and most lived in the villa (70.4%). Nearly, one-third (32.6%) were earning less than 5,000 SAR per month, and others were earning either 5,000-10,000 SAR (16.1%) or 10,001-20,000 SAR (17%). Notably, 38.6% lived with seven to nine family members, and 36.1% lived with four to six. The proportion of patients who had diseases that required an orthopedic visit during the pandemic was 40.9%. Approximately, 20% of the patients visited the orthopedic clinic once during the pandemic, and 12.1% visited twice.

**Table 1:** Sociodemographic characteristics of the patients (N = 635) SAR: Saudi Arabian Riyal.

Study data	N (%)
Age group (years)	
18–25	354 (55.7%)
26–35	174 (27.4%)
36–45	68 (10.7%)
46–55	31 (04.9%)
>55	08 (01.3%)
Gender	
Male	250 (39.4%)
Female	385 (60.6%)
Educational level	
High school or below	199 (31.3%)
Bachelor or higher	436 (68.7%)
Marital status	
Never been married	440 (69.3%)
Been married	195 (30.7%)
Residence region	
Northern region	28 (04.4%)
Eastern region	178 (28.0%)
Central region	264 (41.6%)
Western region	122 (19.2%)
Southern region	43 (06.8%)
Residence area	
Urban	561 (88.3%)
Rural	74 (11.7%)
Type of house	
Villa	447 (70.4%)
Apartment	179 (28.2%)
Others	09 (01.4%)
Monthly income (SAR)	
No income	172 (27.1%)
<5,0000	207 (32.6%)
5,000–10,000	102 (16.1%)
10,001–20,000	108 (17.0%)
>10,000	46 (07.2%)
Number of family members	
1–3	104 (16.4%)
4–6	229 (36.1%)
7–9	245 (38.6%)
≥10	57 (09.0%)
Suffered diseases that requires to go to orthopedic surgeon	
Yes	260 (40.9%)
No	375 (59.1%)
Frequency of visitation to orthopedic clinic	
I did not visit orthopedic clinic yet	290 (45.7%)
Once	125 (19.7%)
Twice	77 (12.1%)
Three times	72 (11.3%)
Four times or more	71 (11.2%)

**Figure 1:**
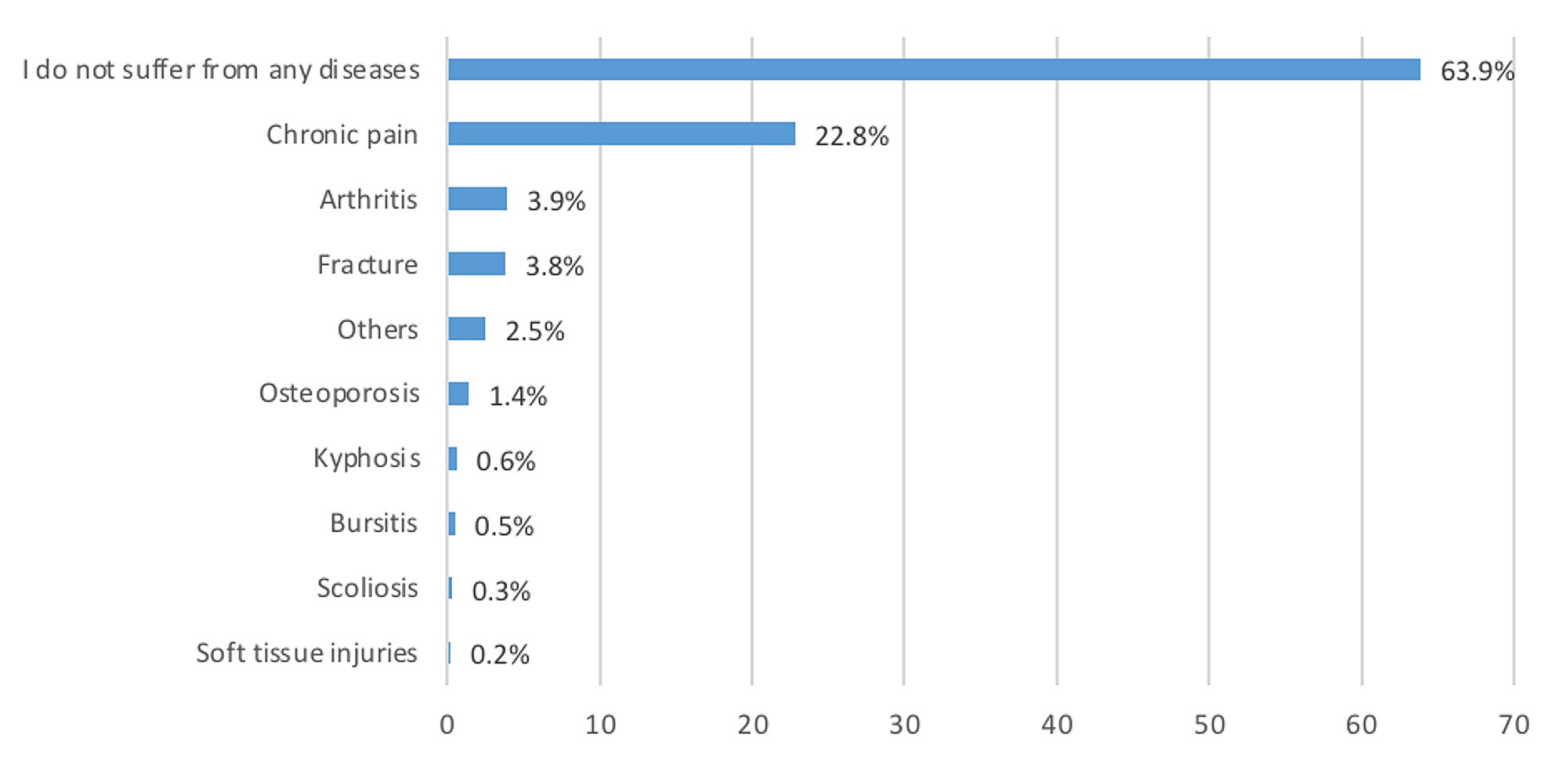
Chronic bone diseases

Figure 1 depicts the chronic bone diseases reported by respondents. The most common conditions were chronic pain (including lower back, shoulder, knee, hand, ankle, and neck pain; 22.8%), arthritis (3.9%), and fracture (3.8%). Soft tissue injuries were the least common (0.2%).

**Figure 2:**
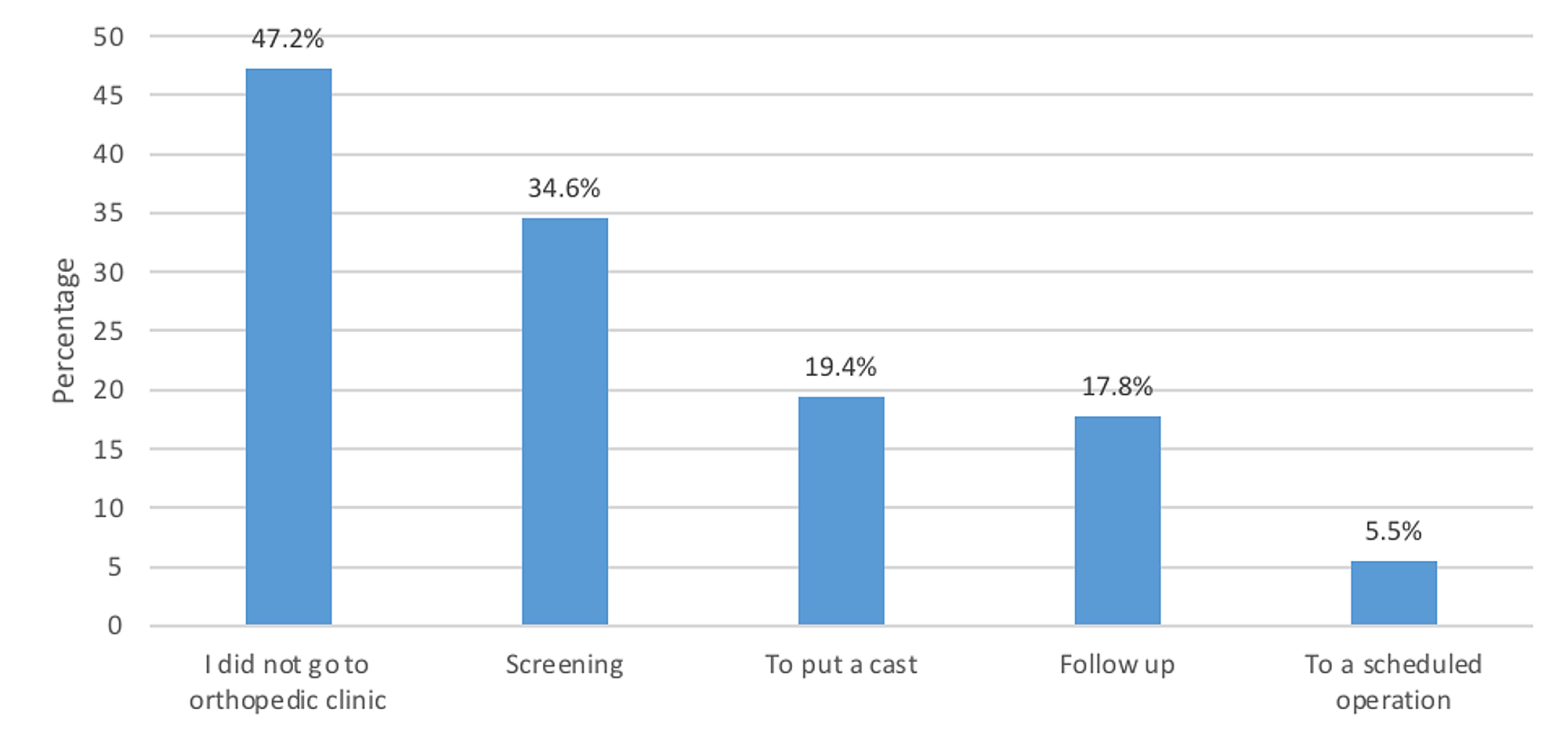
Reason for orthopedic visitation

Figure 2 displays reasons for respondent orthopedic visits. The screening was the most frequent reason (34.6%), followed by casting (19.4%) and follow-up (17.8%).

Table 2 presents patient knowledge about the virtual clinic during the COVID-19 pandemic. The proportions of patients who knew about the virtual clinic and who used a virtual clinic during the COVID-19 pandemic were 47.6% and 30.4%, respectively. The most commonly used method to access a virtual clinic was the Seha app (21.1%). Nearly, half of the respondents (47.1%) were hesitant to go to the hospital because of the pandemic, but 28.8% were able to visit the hospital despite the pandemic situation. The proportion of respondents who believed that the virtual clinic was fully prepared to receive patients was 63%.

**Table 2:** Patient knowledge about virtual clinic (N = 635) *Variable with multiple responses.

Statement	N (%)
Knowledge about the virtual clinic	
Yes	302 (47.6%)
No	333 (52.4%)
Used of the virtual clinic during COVID-19 pandemic	
Yes	193 (30.4%)
No	442 (69.6%)
Applications used during virtual clinic*	
Seha application	134 (21.1%)
Tetamman application	46 (07.2%)
By calling 937	116 (18.3%)
Private hospital application	38 (06.0%)
Others	12 (01.9%)
I did not use the virtual clinic	400 (63.0%)
Do you hesitate to go to the hospital when you are obliged in light of the COVID-19 pandemic?	
Yes	299 (47.1%)
No	154 (24.3%)
Probably	182 (28.7%)
Did you have an outpatient appointment in light of the COVID-19 pandemic?	
No	349 (55.0%)
Yes, I went	183 (28.8%)
Yes, but I did not go	103 (16.2%)
Do you think that the virtual clinic is fully prepared to receive patients?	
Yes	400 (63.0%)
No	235 (37.0%)

**Figure 3:**
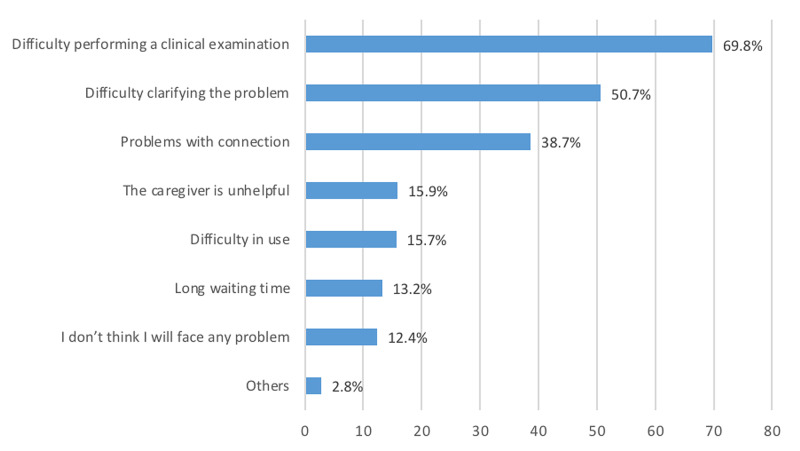
Barriers to using a virtual clinic

As seen in Figure 3, the most commonly reported barrier of the virtual clinic was difficulty performing a clinical examination (69.8%), followed by difficulty clarifying the problem (50.7%) and problems with connection (38.7%). A long waiting time was the least mentioned barrier (13.2%).

**Figure 4:**
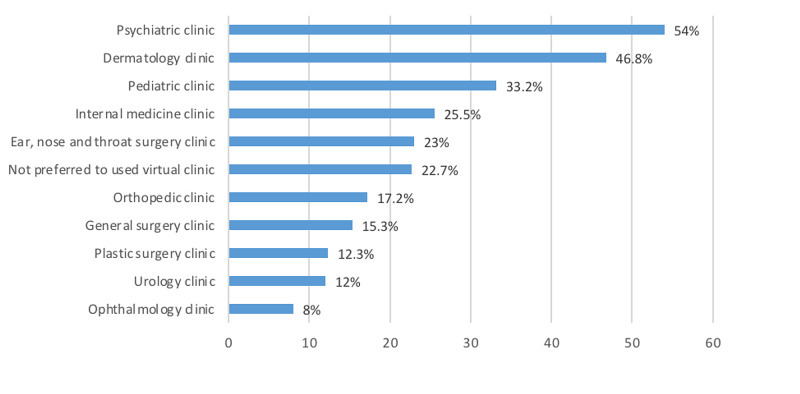
Most common specialties that people are willing to use in a virtual setting

Figure 4 shows the specialties that patients were most willing to use virtually. The psychiatric clinic was most common (54%), followed by the dermatology clinic (46.8%) and the pediatric clinic (33.2%); the ophthalmology clinic was the least common (8%). Notably, only 17.2% of respondents were willing to use a virtual orthopedic clinic.

In the assessment of attitudes (Table 3), patients reported positive attitudes about the statements “Consultation through the virtual clinic is better than going to another city” (strongly agree, 47.7%) and “Virtual clinic can reduce the waiting time and delay” (strongly agree, 42.2%). Conversely, respondent attitudes were negative about the following statements: “I think a doctor would be able to understand me through the virtual clinic video” (strongly agree, 9.8%) and “I prefer to consult a doctor through the virtual clinic than personally in the clinic” (strongly agree, 9.8%).

**Table 3:** Patient attitudes toward virtual clinics (N = 635) SD: strongly disagree, D: disagree, N: neutral, A: agree, SA: strongly agree.

Statement	SD N (%)	D N (%)	N N (%)	A N (%)	SA N (%)
The money that will go to great lengths to see a doctor, such as petrol and other things, will influence my decision on my preference for using a virtual clinic.	76 (12.0%)	184 (29.0%)	151 (23.8%)	26 (19.8%)	98 (15.4%)
I think a doctor would be able to understand me through the virtual clinic video.	44 (06.9%)	151 (23.8%)	178 (28.0%)	200 (31.5%)	62 (09.8%)
I prefer to consult a doctor through the virtual clinic than personally in the clinic.	76 (12.0%)	226 (35.6%)	145 (22.8%)	126 (19.8%)	62 (09.8%)
I feel that the virtual clinic can play a follow-up role to reduce the side effects of treatment.	21 (03.3%)	59 (09.3%)	121 (19.1%)	288 (45.4%)	146 (23.0%)
The virtual clinic can reduce the waiting time and delay.	12 (01.9%)	31 (04.9%)	66 (10.4%)	258 (40.6%)	268 (42.2%)
Consultation through a virtual clinic is better than going to another city.	15 (02.4%)	36 (05.7%)	50 (07.9%)	231 (36.4%)	303 (47.7%)
The unavailability of a specialist in my city could be solved by the virtual clinic.	27 (04.3%)	107 (16.9%)	217 (34.2%)	185 (29.1%)	99 (15.6%)

The descriptive statistical results of the attitudes toward virtual clinics are presented in Table 4. Results showed a mean overall attitude score of 24.4 (standard deviation, 9.9) of 35 points. Negative, neutral, and positive attitudes were classified among 9.9%, 54.3%, and 35.7%, respectively.

**Table 4:** Descriptive statistics of attitudes toward a virtual clinic (N = 635) SD: standard deviation.

Variable	N (%)
Total attitude score (mean ± SD)	24.4 ± 5.29
Level of attitude	
Negative	63 (09.9%)
Neutral	345 (54.3%)
Positive	227 (35.7%)

When attitudes were compared among the sociodemographic characteristics of the patients, younger age (≤25 years vs. >25 years) was significantly associated with a positive attitude (χ^2^=6.068; p=0.048). Compared with respondents who had been married, the never-married participants reported significantly more positive attitudes (χ^2^=6.695; p=0.035). Other sociodemographic variables including gender, educational level, residence region, residence area, type of house, monthly income, number of family members, suffered diseases, orthopedic clinic visits, knowledge about the virtual clinic, and use of a virtual clinic during the COVID-19 pandemic did not show significant differences in attitudes (all p>0.05; Table 5).

**Table 5:** Relationship between the level of attitude and the sociodemographic characteristics of the patients (N = 635) ^§^The p-value was calculated using the chi-square test. **Significance was set at p<0.05. n = number of participants.

Factor	Negative N (%)^(n=63)^	Neutral N (%)^(n=345)^	Positive N (%)^(n=227)^	X^2^	P-value^§^
Age group (years)					
≤25	27 (42.9%)	204 (59.1%)	123 (54.2%)	6.068	0.048**
>25	36 (57.1%)	141 (40.9%)	104 (45.8%)		
Gender					
Male	27 (42.9%)	135 (39.1%)	88 (38.8%)	0.364	0.834
Female	36 (57.1%)	210 (60.9%)	139 (61.2%)		
Educational level					
High school or below	17 (27.0%)	122 (35.4%)	60 (26.4%)	5.691	0.058
Bachelor or higher	46 (73.0%)	223 (64.6%)	167 (73.6%)		
Marital status					
Never been married	41 (65.1%)	254 (73.6%)	145 (63.9%)	6.695	0.035**
Been married	22 (34.9%)	91 (26.4%)	82 (36.1%)		
Residence region					
Northern region	02 (03.2%)	14 (04.1%)	12 (05.3%)	12.910	0.115
Eastern region	17 (27.0%)	92 (26.7%)	69 (30.4%)		
Central region	31 (49.2%)	141 (40.9%)	92 (40.5%)		
Western region	11 (17.5%)	79 (22.9%)	32 (14.1%)		
Southern region	02 (03.2%)	19 (05.5%)	22 (09.7%)		
Residence area					
Urban	59 (93.7%)	303 (87.8%)	199 (87.7%)	1.915	0.384
Rural	04 (06.3%)	42 (12.2%)	28 (12.3%)		
Type of house					
Villa	41 (65.1%)	239 (69.3%)	167 (73.6%)	2.158	0.340
Apartment/others	22 (34.9%)	106 (30.7%)	60 (26.4%)		
Having monthly income					
Yes	48 (76.2%)	240 (69.6%)	175 (77.1%)	4.308	0.116
No	15 (23.8%)	105 (30.4%)	52 (22.9%)		
Number of family members					
1–3	16 (25.4%)	51 (14.8%)	37 (16.3%)	5.504	0.481
4–6	21 (33.3%)	123 (35.7%)	85 (37.4%)		
7–9	22 (34.9%)	136 (39.4%)	87 (38.3%)		
≥10	04 (06.3%)	35 (10.1%)	18 (07.9%)		
Suffered diseases					
Yes	24 (38.1%)	146 (42.3%)	90 (39.6%)	0.639	0.727
No	39 (61.9%)	199 (57.7%)	137 (60.4%)		
Orthopedic clinic visits					
Yes	33 (52.4%)	186 (53.9%)	126 (55.5%)	0.247	0.884
No	30 (47.6%)	159 (46.1%)	101 (44.5%)		
Knowledge about virtual clinic					
Yes	37 (58.7%)	162 (47.0%)	103 (45.4%)	3.637	0.162
No	26 (41.3%)	183 (53.0%)	124 (54.6%)		
Used of virtual clinic during COVID-19 pandemic					
Yes	16 (25.4%)	100 (29.0%)	77 (33.9%)	2.402	0.301
No	47 (74.6%)	245 (150%)	150 (66.1%)		

## Discussion

Telemedicine is used to deliver real-time communication from a distant place between the doctor and the patient through an electronic device (either phone or video call) [5,16]. Telemedicine was identified as an effective way to deliver medical care during the COVID-19 pandemic, and it can be very helpful for the orthopedic specialty [7-10]. Telemedicine has been considered successful for delivering care during the COVID-19 pandemic in Saudi Arabia [17]. The approach can be used in all specialties, even those that require extensive physical examination [16].

The authors have distributed a self-administrated online questionnaire to the Saudi citizens to determine the readiness of the Saudi population to use telemedicine for musculoskeletal care during the COVID-19 pandemic. A total of 635 respondents completed the questionnaire. The mean overall attitude score was 24.4 (standard deviation, 9.9) of 35 points; negative, neutral, and positive attitudes were reported among 9.9%, 54.3%, and 35.7% of respondents, respectively. In our studied sample, positive attitude towards using telemedicine was significantly more associated with those participants who are younger-aged (≤25 years) and had never been married (χ^2^=6.068; p=0.048) and (χ^2^=6.695; p=0.035), respectively.

This study demonstrates the readiness of the Saudi population to use telemedicine for musculoskeletal complaints, and results may contribute to future preparedness for any unavoidable circumstances, such as the COVID-19 pandemic. In this study, the most common musculoskeletal complaint was chronic pain (in particular, lower back pain), which is consistent with previous studies [18,19]. In this study, arthritis was the second most common musculoskeletal complaint, unlike reports from a previously published article [19]. Similar to a previous study, this study identified a lack of knowledge about telemedicine in Saudi Arabia [20]. In our study, the Seha application, followed by the Tetamman application, was the most common application used by the Saudi population. However, another study showed that Tetamman was the most common and that Seha was only the fourth most commonly used application [17].

In our study, 15.7% reported that telemedicine was difficult to use; in another study, the percentage was higher (19.2%) [21]. This study shows that 30.4% of the queried population in Saudi Arabia used the virtual clinic; another Saudi study reported use by 36.6% [21]. However, both studies reported use lower than the published results from the United States, in which 58.2% reported telemedicine use [22]. Almuayqil et al. [23] found that technical issues were the most commonly reported barrier to telemedicine use in a Saudi population, but this result contradicts our findings. In our study, the most common barrier to using telemedicine was difficulty in performing a physical examination. This barrier can be overcome by using evidence-based guidelines with clinical scoring, as reported in a prior study [24]. Some studies also suggest applying certain guidelines to virtual musculoskeletal examinations to overcome this barrier [25,26].

Many researchers have conducted surveys to measure the actual usage of telemedicine in hospital settings. For instance, a survey conducted in China states that 93.8% of tertiary hospitals implemented telemedicine services in various forms [27]. Moreover, Dorsey and Topol [28] reported that telemedicine will be increasingly implemented in different forms, such as mobile stroke units that connect an ambulance with the stroke team to provide images in the field, which saves time in the diagnosis to proceed directly with management. In a study by Gurupur et al. [15], attitudes were positive about virtual clinics, because less waiting and easier accessibility were common.

These results suggest that patients are afraid that the doctor will not be able to understand them or they will not be able to deliver the information to the doctor. When sociodemographic data were reviewed, younger adults were found to be more accepting of telemedicine than older adults; this result is supported by a study conducted in a community in Bangladesh [29]. However, that study suggested that being a woman and having a low economic status increased the difficulties of technology use [29].

However, there are some limitations of this study including recall bias regarding the participants’ use of virtual clinic during the COVID-19 pandemic. Also, we did not combine the questionnaire with structured interviews which could have enhanced the quality of data.

## Conclusions

Our study findings suggest that nearly half of our participants have previous knowledge about the existence of virtual clinics in Saudi Arabia. The most commonly used application for the virtual clinic was Seha. The studied participants showed a moderate level of acceptance to use virtual clinics in Saudi Arabia for musculoskeletal care during the COVID-19 pandemic, but some issues about patient access and understanding of the technology remain unaddressed. Therefore, the authors recommend prospective studies with larger sample sizes and eliminating the possible confounding factors to assure more reliability.

## Appendices

1- Do you agree to fill out the questionnaire?

   Yes

   No

2- Gender:

    Male

    Female

3- Age:

   <18 years

   18-25 years

   26-35 years

   36-45 years

   46-55 years

   >55 years

4- What is the highest educational degree you got?

    High school or below

    Bachelor or higher

5- What is your marital status?

    Never been married

    Been married

6- Nationality:

    Saudi

    Others:

7- What is your residence region?

    Northern region

    Eastern region

    Central region

    Western region

    Southern region

8- Where do you live?

    Urban

    Rural

9- The type of house you live in:

     Villa

     Apartment

     Others:

10- Monthly income:

      No income

      <5,00,00

      5,000-10,000

      10,001-20,000

      >10,000

11- Number of family members:

     1-3

     4-6

     7-9

     ≥10

12- Did you suffer from any diseases/problems that required you to go to the orthopedic surgeon?

      Yes

      No

13- Do you suffer from any of these chronic bone diseases?

      Arthritis

      Bursitis

      Fibromyalgia

      Fractures

      Chronic pain (in the lower back, shoulder, knee, hand, ankle, or neck)

      Soft tissue injuries

      Osteoporosis

      Kyphosis

      Scoliosis

      I do not suffer from any disease

      Others:

14- What is your frequency of visitation to an orthopedic clinic?

      I did not visit the orthopedic clinic yet

      Once

     Twice

     Three times

     Four times or more

15- What is the reason for coming to the orthopedic clinic? You can choose more than one answer.

      To put a cast

      To do a scheduled operation

      Follow up

      Screening

      I didn't go to the orthopedic clinic

Please choose the phrase that matches your opinion if you agree or disagree with the following sentences:

16- The money that will go to great lengths to see a doctor, such as petrol and other things, will influence my decision on my preference for using a virtual clinic.

      I totally agree

      I agree

      Neutral

      I do not agree

      Strongly disagree

17- I think a doctor would be able to understand me through the virtual clinic video.

     I totally agree

     I agree

     Neutral

     I do not agree

     Strongly disagree

18- I prefer to consult a doctor through the virtual clinic rather than personally in the clinic.

     I totally agree

     I agree

     Neutral

     I do not agree

     Strongly disagree

19- I feel that the virtual clinic can play a follow-up role to reduce the side effects of treatment.

      I totally agree

      I agree

      Neutral

      I do not agree

      Strongly disagree

20- Virtual clinics can reduce the waiting time and delay.

       I totally agree

       I agree

       Neutral

       I do not agree

       Strongly disagree

21- Consultation through a virtual clinic is better than going to another city.

      I totally agree

      I agree

      Neutral

      I do not agree

      Strongly disagree

22- The unavailability of a specialist in my city could be solved by a virtual clinic.

       I totally agree

       I agree

       Neutral

       I do not agree

       Strongly disagree

23- Do you have knowledge of the existence of a virtual clinic?

       Yes

       No

24- Did you use the virtual clinic during the COVID-19 pandemic?

      Yes

      No

25- If you answered yes to a previous question, through which program did you use telemedicine?  You can choose more than one option

      Seha app

      Tetamman app

      By calling 937

      Private hospital app

      Others

      I did not use a virtual clinic

26- What are the problems that you may face that hinder your use of the virtual clinic: You can choose more than one option

      Problems with connection

      Difficulty in use

      Long wait time

      Difficulty clarifying the problem

      The caregiver is unhelpful

      I don't think I will face any problem

      Difficulty performing a clinical examination

      Others:

27- Do you hesitate to go to the hospital when you are obliged in light of the COVID-19 pandemic?

      Yes

      No

      Probably

28- Did you have an outpatient appointment in light of the COVID-19 pandemic?

      Yes, I went

      Yes, but I didn't go

      No

29- Which of the following specialties do you think you might use for a virtual clinic (telemedicine) to take medical advice or if you suffer from a problem, God forbid?  You can choose more than one clinic.

      General surgery clinic

      Plastic surgery clinic

      Orthopedic clinic

      Ear, nose, and throat surgery clinic

      Urology clinic

      Internal medicine clinic (such as cardiology clinic, hematology, lung disease clinic)

      Pediatric clinic

      Psychiatric clinic

      Ophthalmology clinic

      Dermatology clinic

      Not preferred to use virtual clinics

30- Do you think that the virtual clinic is fully prepared to receive patients?

      Yes

      No
